# Complement cascade inhibition in geographic atrophy: a review

**DOI:** 10.1038/s41433-021-01765-x

**Published:** 2022-01-09

**Authors:** Dhaval Desai, Pravin U. Dugel

**Affiliations:** Iveric Bio, 1249 South River Road, Suite 107, Cranbury, NJ 08512 USA

**Keywords:** Macular degeneration, Retinal diseases

## Abstract

The pathophysiology of dry age-related macular degeneration (AMD) and specifically geographic atrophy (GA) has been linked to the complement cascade. This cascade is part of the innate immune system and is made up of the classical, alternative, and lectin pathways. The pathways comprise a system of plasma and membrane-associated serum proteins that are activated with identification of a nonself entity. A number of these proteins have been implicated in the development and progression of dry AMD. The three pathways converge at C3 and cascade down through C5, making both of these proteins viable targets for the treatment of dry AMD. In addition, there are a number of complement factors, CFB, CFD, CFH, and CFI, which are potential therapeutic targets as well. Several different complement-directed therapeutics are being studied for the treatment of dry AMD with the hope that one of these approaches will emerge as the first approved treatment for GA.

## 摘要

干性年龄相关性黄斑变性 (AMD), 特别是地图样萎缩 (GA) 的病理生理学与补体级联有关。这种级联反应是先天免疫系统的一部分, 由经典, 替代和凝集素途径构成。这些通路包括一个与血浆和膜相关的血清蛋白系统, 该系统在识别非本体时被激活。其中的许多蛋白与干型AMD的发生和发展有关。这3条通路在C3处汇合, 并通过C5级联, 这两种蛋白为治疗干性AMD的可行靶点。此外, 还有一些补体因子, CFB, CFD, CFH和CFI, 也是潜在的治疗靶点。目前有几种不同的补体导向疗法也正在研究中, 以治疗干性AMD, 希望其中一种成为第一种被批准的GA治疗方法。

## Introduction

### Age-related macular degeneration (AMD) overview

AMD is a progressive retinal disease and the leading cause of central vision loss in the population over age 50 years in developed countries [1]. AMD is classified into two types, dry or nonneovascular AMD and wet or neovascular AMD. In the dry form, loss of photoreceptors and retinal pigment epithelium (RPE) cells in the macula results in atrophy of retinal tissue with the late stage referred to as geographic atrophy (GA). In wet AMD, choroidal neovascularization (CNV) develops under the retina and macula. This form can also continue to atrophy in the late stages, resulting in GA. This suggests that in many AMD patients, regardless of whether they have the dry or the wet form, the final anatomic outcome leading to loss of vision is GA. While wet AMD accounts for much of the vision loss due to AMD, it comprises only about 10% of the entire AMD population [2]. The 90% of AMD patients who suffer from dry AMD can also have devastating vision loss when end-stage GA develops.

### Dry AMD

Dry AMD arises from defects in the RPE leading to the formation of drusen, a build-up of protein and lipid aggregates, on and around the macula. Drusen deposition occurs between Bruch’s membrane and the RPE and is thought to be a result of structural defects in the RPE [3]. However, knowledge of drusen composition, origination, and formation in the macula is still not completely understood [4]. While small drusen alone are not the direct cause of AMD, intermediate and large drusen lead to increased risk of AMD progression [5]. Because of this, drusen size classification has become increasingly important at initial diagnosis. Currently, clinicians classify drusen into three groups according to size: small (<63 µm diameter), intermediate (>63 µm but ≤ 125 µm diameter), and large (>125 µm diameter) [1]. Since drusen that fall into the “small” size designation do not significantly correlate with AMD, the term “drupelets” has been used to differentiate these drusen that are often a normal aging change. Intermediate and large drusen correlate directly with AMD as determined by the Age-Related Eye Disease Study (AREDS) [1].

Within dry AMD, GA is an advanced form, severely affecting vision and often threatening complete vision loss in an estimated 1.5 million individuals in the =USA and up to 5 million worldwide [6]. Trends project GA prevalence to rise in the coming years, with estimates of 18.57 million cases globally by the year 2040 [7]. Progression to GA is irreversible, and there are currently no approved treatments.

## Dry AMD progression and monitoring

### GA progression

GA formation in one eye highly suggests that GA will, at some point, affect the other eye [8]. While atrophy may not occur simultaneously, it has been estimated that the median time between eyes progressing to GA is 7 years, based on AREDS [8]. In AREDS, there were 686 total participants, none of whom had neovascularization or GA at baseline in either eye. Of these participants, 209 participants were diagnosed with bilateral GA at follow-up visits. The other 477 participants were not affected by GA in their second eye. In these patients, it was found that the median time between the development of GA in one eye and the development of GA in the second eye thereafter is 7 years (95% CI, 6.0–10.0) [8]. Moreover, bilateral GA strongly indicates an overall accelerated progression of atrophy.

Lesion location is also an important indicator in GA progression. Extrafoveal lesions progress more rapidly than foveal lesions, which may help clinicians evaluate the probability of disease progression in order to better educate patients [8]. The GA Study showed that subjects with foveal-sparing GA at baseline had 2.8-fold faster lesion progression toward the periphery than toward the fovea [8]. It has been estimated that the median time of the progression of GA from initial discovery to foveal center involvement is 2.5 years [8]. Lesion size is another standard metric used to assess GA severity and rate of progression. Previous studies have described lesion growth rates from 0.53 to 2.6 mm^2^/year, varying based on both individual specific factors and nonspecific (or external) factors [8]. In addition, lesion enlargement and GA progression leads to a decline in visual function and overall worsening of disease severity. In a prospective case series by Sunness et al., atrophic enlargement and visual acuity (VA) loss was examined in patients with GA [9]. At baseline, eyes with larger areas of atrophy had a poorer median VA [9]. In this study, 31% of all study eyes had a three-line VA loss from baseline after 2 years, and 53% had a three-line loss from baseline after 4 years. Of nine patients who had GA in one eye (and no GA in the fellow eye), 22% developed GA in the fellow eye at 2 years [9].

### GA monitoring

Lesion size is the main measure of disease progression in the eyes of patients with GA. Progression of GA is also based on the baseline lesion size, so measuring lesion size at baseline and as the disease progresses is imperative [8].

Technology to diagnose and monitor GA has continued to advance. For many years, color fundus photography was the gold standard for monitoring progression of GA, but monitoring by this technique is often imprecise and lacks the elegance of newer modalities [8]. Spectral-domain optical coherence tomography (SD-OCT) and polarization-sensitive SD-OCT (PS-OCT) can be useful in monitoring disease progression as well. PS-OCT can detect the lesion size in patients with GA and accurately quantify the areas of atrophy within the RPE [8]. OCT classification of lesion severity has not been standardized within the retina community, however, making consensus analyses difficult [8].

A system for AMD classification as it relates to degree of atrophy, measured via OCT, has been proposed as part of the Classification of Atrophy Meetings (CAM) group [10]. Here, the group notes that since photoreceptor atrophy may occur without RPE atrophy, various stages, and therefore designations, of atrophy as it relates to retinal disease progression should be standardized. Notably, the term “complete RPE and outer retinal atrophy” was offered to aid in defining the most severe cases of atrophy. For these classifications, other imaging modalities offered complementary methods to support the group’s proposals.

Despite the CAM group’s suggestions, fundus autofluorescence (FAF) is the gold standard short-wavelength imaging method used to report lesion growth in clinical trials. Utilizing blue light (488 nm wavelength), FAF can detect a natural fluorescing retinal pigment, lipofuscin, and produce retinal images useful in understanding GA pathophysiology [11]. These intrinsic fluorescing patterns offer greater information over standard fundus photography and OCT, making FAF a key tool for diagnosing and tracking GA progression [12]. The pattern of the lesions at the junctional zone in FAF is a prime indicator of the severity of GA in a patient. The classification of lesion pattern is broken down into none, focal, banded, patchy, or diffuse [8]. Individuals with banded or diffuse patterns show greater GA progression than individuals with a focal pattern or no pattern at all.

Other imaging modalities, such as near-infrared reflectance imaging (NIR), flood-illuminated widefield FAF, and green-wavelength widefield FAF, are also available to determine lesion size [8]. These additional imaging modalities are valuable for adjunctive use when foveal integrity is questioned while using only short-wavelength FAF. NIR lesions appear brighter, which assists in determining foveal lesion boundaries. NIR may show variability in results depending on the subfoveal choroidal thickness [8].

GA lesion size and pattern are the benchmark quantitative measurements in evaluating GA progression, but assessing visual function is important as well. Therefore, repeated VA testing over time can also help monitor disease progression. Best-corrected VA (BCVA) and low-luminance VA (LLVA) are considered the gold standards. LLVA may identify visual changes in response to AMD earlier than BCVA [13]. Similarly, a decrease in reading speed may also indicate lesion growth into the central macula and has been utilized as a metric to assess disease progression [14].

Finally, microperimetry may be used as a complement to VA measurements. Microperimetry measures retinal movements in real-time via scanning laser ophthalmoscope and maps retinal sensitivity on a fundus image. Microperimetry can identify macular pathology prior to centralized vision loss and is a noninvasive, evolving technique of interest in AMD diagnosis [15].

These tools for the diagnosis and monitoring of GA are increasingly important as research leads to improved understanding of potential underlying disease mechanisms.

### Risk factors for GA

Several prognostic factors influence GA risk including age, genetics, and various external factors such as smoking. Age is the most common predictor of GA, with multiple studies confirming that GA severity increases with age [3, 6, 16–18]. GA is generally not common in individuals under age 50 years and, compared to individuals between ages 65 and 74, the risk of GA development increases more than threefold for those over age 75 years. In the USA, more than 30% of people over 85 years of age develop GA [3].

Various biological pathways play roles in AMD and progression to GA, with genetic factors a key determinant. Several genome-wide association studies indicate two main genetic loci with links to AMD and GA formation: 1q31 and 10q26, which correspond to the complement pathway and high-temperature requirement A serine peptidase 1/age-related maculopathy susceptibility 2 (HtrA1/ARMS2) gene polymorphisms, respectively [19]. HtrA1 is a serine protease ubiquitously secreted by the RPE, and studies have shown that overexpression of HtrA1 increased defects in both the RPE and Bruch’s membrane as well as increased risk for CNV [20–22]. In this review, our main focus is the complement pathway, which comprises a cascade of enzymatic functions within the immune system response. In dry macular degeneration, complements C3 and C5 play a critical role. There are also rare mutations in complement factor H (CFH) and complement factor I (CFI), which potentially dysregulate the cellular pathway leading to AMD as well.

## The complement cascade

### Understanding the innate immune system

The mammalian immune system is remarkably complex and comprises two immune processes—adaptive and innate. These two immune processes have a function in host defense, enabling the efficient detection and elimination of pathogens to protect the host. The adaptive immune system ensures the host stays safe from pathogens chronically; it helps the host create long-lived immunological memories to prevent reinfection [23]. The innate immune system, in contrast, provides immediate and nonspecific responses to the introduction of any pathogen [24]. This is the first line of defense against any infection (nonself pathogen) or internal tissue injury. Innate immunity is a sophisticated protective network of serum proteins, cell-surface regulators, and receptors and is vital to maintaining a healthy tissue microenvironment [25].

### Complement cascade

**Fig. 1:**
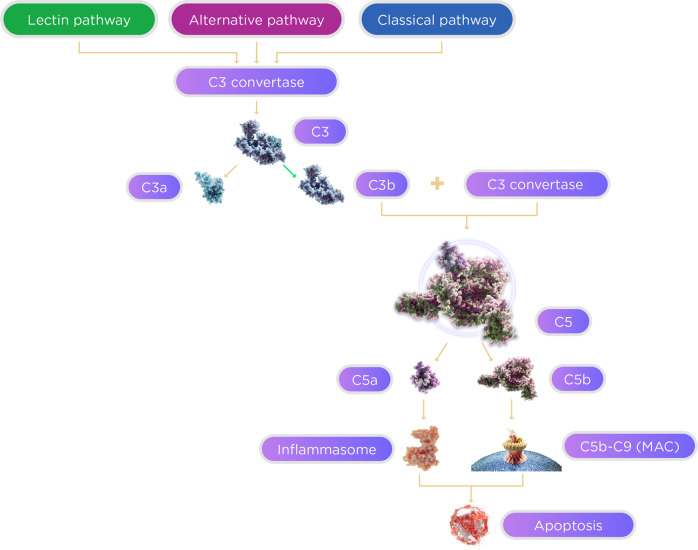
The complement cascade. The complement cascade is a highly conserved component of the innate immune system consisting of serum and membrane-bound proteins. This proteolytic cascade is activated through 3 pathways: the classical, alternative, and lectin. The activation pathways converge at C3 convertase, activation of which cleaves C3 into C3a and C3b subunits. As the cascade continues, C5 convertase cleaves C5, leading to inflammasome and MAC formation, ultimately resulting in apoptosis.

The innate immune system consists of multiple effector mechanisms, one of which is the complement system (Fig. 1). The complement system plays a crucial role in the innate immune response against pathogens; it recognizes, tags, and eliminates any foreign particles [25]. Overall, the complement cascade is an intricate system of plasma and membrane-associated serum proteins. At baseline, the complement system is inactive. Infectious organisms, injury to tissue, and other nonself molecules are identified as “danger signals” that elicit an efficient and regulated inflammatory and cytolytic immune response from proteins within the complement system [26]. There is evidence that there may be other novel mechanisms of complement activation, but the three pathways, classical, lectin, and alternative, play a large role in the host’s response to any foreign pathogen [23].

Once activated, these pathway mechanisms synergistically protect the host from pathogens within the innate immune system [23]. All three complement cascade pathways lead to either the recruitment and activation of inflammasomes, which are proinflammatory molecules, or the formation of membrane attack complex (MAC) (C5b-9) [27].

The first of the three mechanisms, the classical pathway, is activated by immune complex deposits, such as IgG or IgM [26]. During the invasion of pathogens and the creation of antigen–antibody complexes as a result of the host’s immune response, the Fc portion of the antibody that contains the complement binding site gets exposed. This exposure jump-starts the complement cascade by activating pattern recognition molecules (PRMs). C1q, the main component of the C1 complex and the singular PRM of the complement cascade, attaches to the antibody site, which then initiates a conformational change within the C1 complex. This leads to the autocatalytic activation of C1r and C1s, serine protease units that cleave PRMs C4 and C2 into the smaller C4b and C2a fragments and the larger C4a and C2b fragments [25]. C1s specifically form the larger complex, C4bC2a, which then gains the ability to cleave C3; due to its dual functionality, this large complex gets renamed C3 convertase. The classical pathway merges with the alternative and lectin pathways at this point, and all three pathways follow an identical downstream route.

The activation mechanism and initiation of the alternative pathway is distinct from that of the classical and lectin pathways. The alternative pathway is activated by the spontaneous hydrolysis of C3 in plasma and is an antibody-independent route. This path also engages proteins, namely factor B (CFB), factor D (CFD), factor H (CFH), and properdin among others. Hydrolyzed C3 becomes C3(H_2_O), a C3b analog. C3(H_2_O) then binds to CFB, which calls on CFD to transform the complex into C3 convertase [23, 26].

The third effector arm, the lectin pathway, is activated by nonself recognition. Germline-encoded pattern recognition receptors (PRRs), specifically mannose-binding lectin (MBL) and ficolins, identify nonself molecules and activate C2 and C4 downstream. These PRRs specifically focus on identifying selected highly conserved structures, pathogen-associated molecular patterns. Similar to the role of C1q in the classical pathway, MBL contains many serine proteases which are similar, but not identical, to C1r and C1s. These proteases lead to the cleavage of C2 and C4, creating C3 convertase [28].

After creating C3 convertase through one of the three effector arms, C3 convertase then cleaves and activates C3 into C3a (an anaphylatoxin) and C3b (an opsonin). This is the point within the complement cascade, as previously mentioned, where all three complement pathways converge. C3b undergoes binding activity, which essentially tags the pathogen as a foreign entity. This leads to the formation of C5 convertase, C3bBbC3b (for the alternative pathway) and C4bC2aC3b (for the classical and lectin pathways), which causes further complement activation. The terminal phase of the complement system is initiated by C5. The C5 convertase then cleaves C5 into C5a and C5b. C5b then interacts with C6, which initiates the process toward the formation of MAC (C5b-9). The consequent binding of C7 to C5b6 forms a stable complex, C5b-7, which binds with C8, forming C5b-8. C5b-8 promotes the binding and polymerization of the numerous molecules of C9. This entire process then leads to the formation of MAC. Afterward, the final step is the insertion of MAC (C5b-9) into the cell membrane of the foreign particle, leading to cell death [28].

In addition to the formation and infiltration of MAC, the formation of inflammasomes is triggered by the complement cascade and ultimately activates proinflammatory cytokines [29]. The inflammasome is composed of a nucleotide-binding domain and a leucine-rich protein, known as Nod-like receptor (NLR) protein. The assembly of the inflammasome protein itself triggers the activation of caspase-1, which induces the maturation and activation of the cytokine IL-1β [27]. NLRP3 is the most well-characterized inflammasome [29].

The complement cascade is helpful in containing and responding to foreign molecules, but the products of complement activation, specifically C3a and C5a, are known to be effective chemoattractants and anaphylatoxins. Accumulation of C3 and its fragments within the cascade in the damaged tissue helps in the two other response mechanisms of the complement cascade within the innate immune system—opsonization and phagocytosis [25]. C3a and C5a are extremely potent inflammatory mediators within the complement cascade. These anaphylatoxins are known to regulate both immune and nonimmune functions. These functions include, but are not limited to, vasodilation, histamine release, chemoattraction, tissue regeneration, and proinflammatory cytokine production. It has also been hypothesized that C3a and C5a serve as activation signals that trigger the formation of inflammasomes and mediators of inflammatory cytokines [29].

## Targeting the complement cascade for the management of GA

### Complement as a contributor in GA

Multiple studies have implicated complement activation as a key component in the development and progression of GA. Complement proteins, age-dependent increases in the upregulation of complement genes and related accumulation of MAC, and inflammatory cytokines and chemokines found in the retina have supported this hypothesis [30]. Abnormalities in the function of the proteins associated with the complement system lead to an imbalance in homeostasis, often resulting in damage to healthy tissue. AMD, and consequently GA, has recently been added to the long list of diseases associated with such abnormalities within the complement system. Naturally, many protein changes can occur due to genetic dysfunction. The deficiency of CFH, a major regulator in the alternative pathway, is known to lead to uncontrollable activation of the complement cascade via the alternative pathway. An overall deficiency in CFH and/or any inhibition of CFH would also lead to overactivation [31]. Currently, there are not enough clinical data to provide insight into the specific locations in the complement cascade that affect the development and progression of GA. Upregulation of CFH, CFB, CFD, CF1, C1, C3, and C5 are thought to present as risk factors for both the development and progression of GA [30, 32, 33]. The activation of components in the complement pathway and their resulting biological function are summarized in Table 1 [32, 34–42].

**Table 1:** Complement factor activation and resulting biological functions.

Factor	Result in pathway	Biological function
Complement C1 [32, 34]	Self-activates; recognition protein C1q binds to catalytic subunit C1r	Activates complement cascade via classical pathway
Complement C3 [32, 35, 36]	Cleaved into C3a and C3b via C3 convertase	C3b, together with C3 convertase, cleaves C5: beginning of pathway, can affect all components downstream
Complement C5 [35–37]	Cleaved into C5a and C5b via C3b and C3 convertase	MAC (C5b-C9) assembly, proinflammatory response; immunomodulatory and effector capabilities
Complement factor H [38]	Binds to self-markers and prevents complement activation	Anti-inflammatory response; genetic mutations are associated with complement dysregulation; complement cascade is overactive with CFH absence
Complement factor B [39]	Involved in activation of C3	Regulator of alternative pathway; fluid created by hepatocytes and is expressed in retina in mouse models
Complement factor D [40]	Cleaves CFB via conformational change elicited by C3b	Component of alternative pathway
Complement factor I [41, 42]	Mediates C3 cleavage	Predominantly expressed in RPE and photoreceptors

**Fig. 2:**
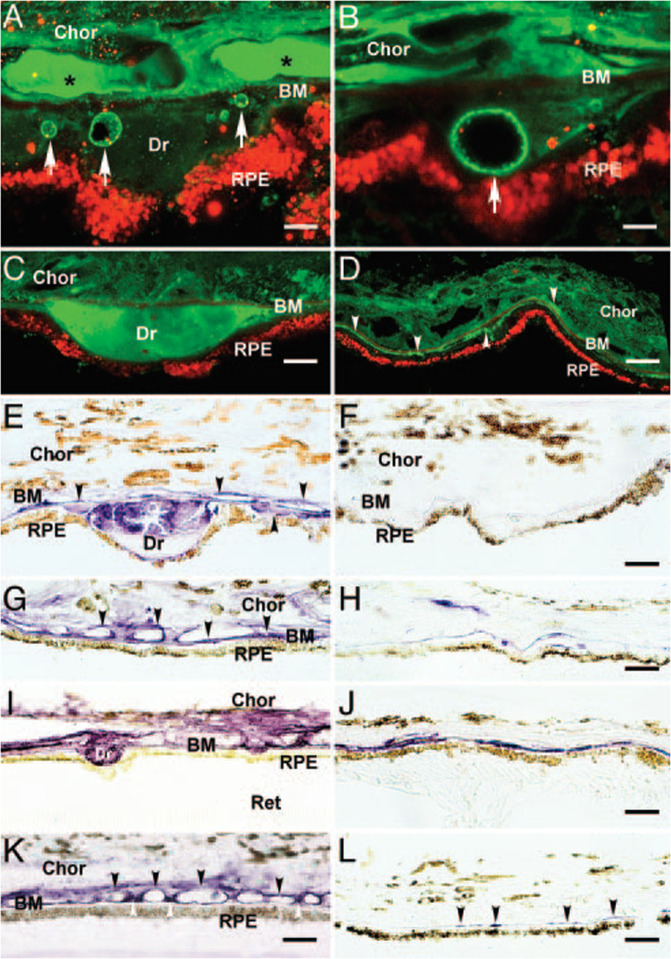
Evidence of complement activity in the formation of drusen [30]. Figure used with permission: Copyright (2005) National Academy of Sciences, USA.

In addition, there is evidence that overactivity of the complement pathway leads to the formation of drusen. Drusen are known to be hallmark lesions in AMD and specifically in GA. Moreover, CFH (HF1/CFH), a natural inhibitor of the complement cascade, is found accumulated in drusen (Fig. 2) [33].

There are many retinal diseases associated with the complement system based on the compelling evidence that shows mRNA complement components in the RPE and choroid of the retinal layers. For each disease, different components of the complement cascade are upregulated and/or downregulated, resulting in variations in levels of complement proteins. Some retinal diseases thought to be involved with the complement cascade are uveoretinitis, diabetic retinopathy, and glaucoma [31].

## Current therapeutic targets and therapies in GA

It is unfortunate that, although patients with dry AMD comprise 90% of the overall AMD population [2], dry AMD has proven to be markedly more recalcitrant to identification of effective treatments compared to wet AMD. As a result, there are no FDA-approved treatments for dry AMD. The therapeutic candidates that currently show the greatest promise for success, however, are those that target the complement system [43].

### Current therapeutic targets

While research has focused on the complement system as a potential way to target dry AMD, the specific pathway—classical, alternative, or lectin—has not yet been definitively determined. Table 2 [44, 45] summarizes current approaches targeting elements of the alternative pathway including CFB, CFD, CFH, and CFI as well as strategies that focus on the common pathway components C3 and C5. The lectin pathway may also have a potential target, MBL-associated serine protease (MASP), which is an enzyme in the lectin pathway of the complement system [46]. MASP-2 is of particular interest in that it has the ability to cleave C2 and C4, although to date there have been no clinical studies initiated in dry AMD [45].

**Table 2:** Potential complement pathway therapeutic targets for dry AMD [35, 44, 45].

Target	Molecule(s)	Company	Type/MOA	Pathway	Comments
Complement factor B	IONIS-FB-L_Rx_	Roche/Ionis	Subcutaneous ligand-conjugated (LICA) antisense therapy	Alternative pathway	Phase II GOLDEN trial ongoing
Complement factor I	GT005	Gyroscope	Subretinal AAV2 vector; gene therapy designed to induce expression of CFI	Alternative pathway	Phase I/II FOCUS trial showed GT005 to be well tolerated; phase II EXPLORE and HORIZON studies are ongoing
Complement factor H	GEM103	Gemini	IVT recombinant human CFH	Alternative pathway	Phase 2a ReGAtta trial enrollment complete
Complement factor I	GEM104	Gemini	IVT full-length recombinant human CFI	Alternative pathway	In preclinical development
Complement factor D	ALXN2040	Alexion/AstraZeneca	Oral factor D inhibitor	Alternative pathway	IND application submitted; phase II study planned
Complement C3	NGM621	NGM Bio	IVT humanized IgG1 monoclonal antibody	Classical pathway	Phase II CATALINA study currently recruiting
Complement C3	Pegcetacoplan	Apellis	IVT cyclic peptide-bound polyethylene glycol polymer	Classical pathway	Phase II FILLY trial showed reduction of GA growth; phase III DERBY and OAKS trials ongoing
Complement C5	Avacincaptad pegol	Iveric Bio	IVT pegylated RNA aptamer	Classical pathway	Phase II GATHER1 trial showed decrease in GA lesion size; phase III GATHER2 trial ongoing

As seen above in Fig. 1, all three pathways of the complement system converge at C3, making it a much-studied target. C3 has a role in the amplification loop of the pathway, generation of anaphylatoxins, and formation of MAC, so targeting C3 decreases all of these. Once the pathway converges at C3, a vital protein within the complement system, C3 convertase then cleaves C3. C3 contains an internal thioester bond, which is activated once C3 is cleaved into C3a and C3b, and the cascade continues downstream. The overall regulation of C3 cleavage is important to monitor, and dysregulation within this process may lead to the initiation and progression of disease. By targeting the complement cascade at this level, all the downstream effects are halted or minimized [41].

**Fig. 3:**
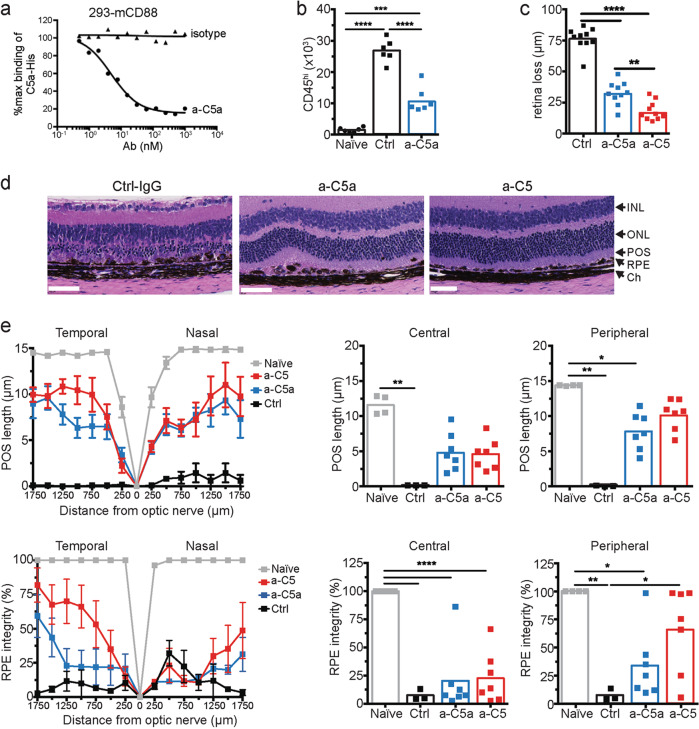
Blocking C5a demonstrated a 60% reduction in retina loss in a mouse model. Blocking C5 activation, which prevents the formation of both C5a and C5b, improved retinal protection over blocking C5a alone. [34]. C5a is required for photoreceptor loss. **a** Anti-C5a (a-C5a) inhibits C5a binding to C5aR on the surface of 293 cells transfected with C5aR (CD88). **b** Flow cytometry analysis of CD11b+CD45hi mononuclear phagocytes in the neural retina of mice treated with control or C5a neutralizing Abs 3 days following NaIO_3_ administration, *n* = 6. **c** Effect of C5a and C5 blocking Abs or isotype control Abs (Ctrl) on retina degeneration as measured by SD-OCT 7 days after NaIO3 administration. **d** Representative H&E stained sections of the central retina 7 days after NaIO3 administration. **e** POS length and RPE integrity measured in horizontal sections along the temporal-nasal axis of the mouse retina. Error bars indicate ±SEM, *n* = 3–4 naive and Ctrl, *n* = 7 a-C5a and a-C5. Naive = not NaIO3 treated. Scale bar = 10 μm. Central is 500 μm from optic nerve and periphery is 500–1750 μm. Experiments were repeated at least twice with similar results. *P < 0.05, **P < 0.01, ***P < 0.001, ****P < 0.0001 by one-way ANOVA with Tukey’s multiple comparisons test. INL inner nuclear layer, ONL outer nuclear layer, POS photoreceptor outer segments, RPE retinal pigmented epithelia, Ch choroid. Figure used under a Creative Commons Attribution 4.0 International License (http://creativecommons.org/licenses/by/4.0/; no changes were made).

C5 has emerged as an attractive target to try to preserve the positive functions for which C3 is responsible. C5, highlighted previously, is downstream from C3 and where the recruitment of inflammasomes and the formation of MAC (C5b-9) occurs. In addition, it is a protein that plays a demonstrated role in the pathogenesis of AMD due to its presence in drusen. C5a is also shown to be elevated in the peripheral blood levels of patients diagnosed with AMD. C5a, specifically, has many proinflammatory and immunomodulatory functions within the complement cascade [43]. Laser-induced CNV mouse models have also shown that C5a is upregulated in the RPE and choroid [46]. SD-OCT imaging of mice showed that retinal loss decreased by ~60% by treating with a-C5a inhibitor. The imaging showed that inhibiting C5a could potentially provide retinal protection (Fig. 3) [45]. C5 activation is truly the first step in the process of the assembly of MAC. This activation of C5 leads to pathological events processes in the inflammatory response of the innate immune system. As mentioned, all components of C5 have immunomodulatory and effector capabilities [43].

When C3 is cleaved into C3a and C3b, C3a is known to reduce cytokine release, thereby acting in an anti-inflammatory capacity. This anti-inflammatory benefit may be lost if the complement cascade is blocked at C3. Another role attributed to C3 is as an anti-infective. Again, blocking the cascade at C3 may result in increased risk for infection [47]. By going farther downstream in the complement cascade and blocking C5, the host defense mechanisms conferred by C3 may be preserved while still blocking the recruitment of inflammasomes and formation of MAC [41].

### Therapies for dry AMD and GA

A number of common and rare genetic variants in the complement system have been linked to the occurrence of dry AMD, suggesting that dysfunction of the complement system may contribute to the drusen formation and GA that serve as hallmarks of dry AMD [48]. Initial investigations into the complement system inhibitor class proved unfruitful. In 2018, lampalizumab (Genentech, Inc., South San Francisco, CA), an intravitreal (IVT) monoclonal antibody fragment that inhibits CFD, was shown to be ineffective at slowing GA lesion growth when administered every 4 or 6 weeks in patients with dry AMD in the phase III CHROMA and SPECTRI trials [49]. In these trials, GA lesion size progression remained substantial across all patient groups (lampalizumab groups and sham groups) with a consistent mean of 2 mm^2^ of growth over the 48-week trial period. These studies did not include subjects with lesions on the smaller or larger side, subjects with unilateral GA, subjects whose eyes had current or prior CNV, subjects who had GA that resulted from causes other than AMD, and subjects at earlier disease stages [49]. All of these are potential limitations of CHROMA and SPECTRI. The main limitation, however, was that the phase III study design was based on a retrospective subgroup analysis [49]. The same core clinical trial design should have been utilized in the phase III trials as was used in the phase II trials to increase the odds of trial success.

Other trials have failed as well, including LFG316, a fully human, high-affinity anti-C5 antibody (Novartis AG, Basel, Switzerland); eculizumab, a humanized anti-C5 antibody (Alexion Pharmaceuticals Inc., Boston, MA); and CLG561, a humanized antibody fragment targeting properdin (Novartis AG, Basel, Switzerland and Alcon Inc., Fort Worth, TX) [50].

Despite these setbacks, recent investigations into complement system inhibitors for GA have proven more promising. In 2017, it was shown in the phase II FILLY trial that pegcetacoplan (Apellis Pharmaceuticals, Waltham, MA), an IVT cyclic peptide-bound polyethylene glycol polymer that inhibits C3 and C3b, significantly reduced the rate of GA lesion growth in patients with dry AMD compared to sham treatment [51]. Overall, pegcetacoplan treatment reduced GA growth rates by 29% (*P* = 0.008) when administered monthly and 20% (*P* = 0.067) when administered every other month compared to sham. Although the effect was not statistically significant in the first 6-month period, the effect was greater in the second 6 months of the study, with reductions of 45% (*P* = 0.0004) and 33% (*P* = 0.009) in the monthly and every other month groups, respectively [51]. There was a higher incidence of neovascularization in the eyes treated with pegcetacoplan. Overall, 18 out of 86 total eyes (20.9%; 95% CI, 12.9–31.0) in the monthly dosing group and 7 out of 79 total eyes (8.9%; 95% CI, 3.6–17.4) in the EOM dosing group presented with neovascularization [51].

In 2019, findings were reported from the phase II/III GATHER1 trial of avacincaptad pegol (ACP) (Iveric Bio, New York, NY), an IVT pegylated RNA aptamer that inhibits complement C5 [52]. In this trial, change in GA progression was measured via FAF at 6 and 12 months and compared to baseline measurements. In part 1 of this trial, participants received either 1 or 2 mg of ACP or sham injection given as a single injection. In part 2 of this trial, subjects received either 2 mg (one injection and one sham procedure) or 4 mg (two injections) of ACP or sham (given as two sham procedures). This two-part approach was done to maintain the double-masking throughout parts 1 and 2 of the trial. In the prespecified primary endpoint analysis, the 2-mg groups from parts 1 and 2 were combined, as were the sham groups.

The 4-mg group was compared to the part 2 sham only.

**Fig. 4:**
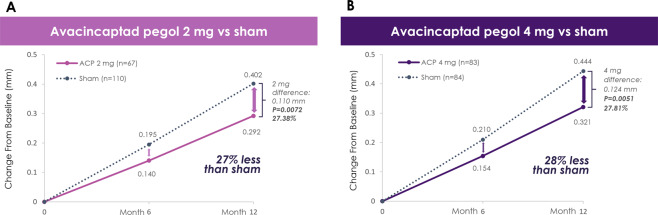
Mean change from baseline in square root GA lesion area over 12 months. **A**ACP 2 mg **B** ACP 4 mg.

Mean change in square-root GA lesion area at the 12-month primary endpoint was 27.8% (*P* = 0.0051) and 27.4% (*P* = 0.0072) less than sham injection in the 2-mg and 4-mg dosage groups, respectively (Fig. 4) [52].

Pegcetacoplan and ACP are currently under ongoing further evaluation in larger-scale clinical trials. Refer again to Table 2 for a summary of these and other therapeutic agents in development targeting the complement pathway for dry AMD [44, 45].

## Overcoming the challenges of dry AMD treatment

One challenge of the study of GA in dry AMD is the lack of suitable animal models, which has constrained characterization of dry AMD to clinical features observed across genetically and phenotypically heterogeneous patient populations. Interestingly, a mouse model of laser-induced retinal damage appears to mimic pertinent clinical features of human GA [53]. Animal models such as this have the potential to accelerate research into dry AMD pathogenesis and serve as useful experimental systems for in vivo evaluation of novel therapeutics for dry AMD.

Ideally, finding therapeutics that reduce the size of GA lesions or reverse progression of dry AMD would be our therapeutic goals, but treatments that slower lesion growth and blunt disease progression represent a significant advancement, especially since these patients currently have no treatment options. In addition, more thorough analysis of current treatment methodologies may provide deeper understanding in determining the optimal stage of therapeutic intervention. Currently, there is limited understanding in this area, but reexamination of current practices (i.e., recruitment of patients with disease that is too far advanced) may be prudent in maximizing potential of pharmacologic treatments.

Well-documented challenges such as noncompliance and undertreatment for IVT antivascular endothelial growth factor drugs used in wet AMD treatment are likely to apply to any potential GA treatment [54]. Alternative delivery methods for dry AMD treatments that circumvent the need for frequent IVT injections may offer solutions to some of these challenges. Overall, the increasing sophistication of ocular drug delivery methods has the potential to propel innovation throughout the growing dry AMD space.

Even with the lack of well-developed animal models for preclinical work, the challenge of not only slowing growth but reversing progression of GA lesions, and the possible need for alternate delivery strategies, there is still hope on the horizon. With a more robust understanding of the complement system, scientists have identified a number of potential targets that show promise. Whether the answer to effective treatment of GA is targeting C1, C3, or C5, the science is encouraging, and a number of therapeutics is being developed.

After not having any treatments for GA secondary to dry AMD beyond being able to recommend vitamins and supplements or make a referral for low vision services, the number of treatments in development is nothing short of extraordinary. In a patient population for whom options have been nonexistent, being able to consider enrollment into one of a number of clinical trials is encouraging. As these GA trials progress and results are shared and published, an even greater understanding will be developed of the role of the complement system in the pathogenesis of dry AMD and specifically GA. With understanding comes progress and with progress comes hope: hope for physicians, hope for family members and caregivers, and, most of all, hope for dry AMD patients with GA.
